# La grossesse ovarienne: un challenge échographique

**DOI:** 10.11604/pamj.2019.33.196.14354

**Published:** 2019-07-12

**Authors:** Amel Achour Jenayah, Mohamed Waheb Abdallah

**Affiliations:** 1 Service “A” de Gynécologie-Obstétrique, Centre de Maternité et de Néonatologie de Tunis, Tunis, Tunisie

**Keywords:** Grossesse ectopique, échographie, cœlioscopie, méthotrexate, Ectopic pregnancy, scan, laparoscopy, methotrexate

## Abstract

Ovarian pregnancy (OP) is rare: it accounts for 3.2% of ectopic pregnancies. We here report the case of a 36-year old patient gravida 3, para 2. The only risk factor for ectopic pregnancy found in this patient was a history of intrauterine contraceptive device (IUCD) use. Clinical symptoms were dominated by the occurrence of pelvic pain at 5-weeks of amenorrhoea. At the initial visit to the Emergency Department ß-Human chorionic gonadotropin (HCG) was 2134 IU/L. Preoperative diagnosis of ovarian pregnancy was suspected due to a rounded image measuring 26 mm with double echogenic crown visible on the left ovary without associated free fluid in the pouch of Douglas on ultrasound. Treatment was based on coelioscopy with resection of the ectopic chorionic villi in the left ovary. Medical treatment was based on intramuscular injection of methotrexate 1 mg/kg body weight in the immediate post-operative period because of the risk of a little residual of trophoblastic material. The diagnosis was confirmed by the histological study of the surgical specimen. The postoperative course was uneventful

## Image en médecine

La grossesse ovarienne (GO) est rare: 3,2% des grossesses extra-utérines. Notre patiente était âgée de 36 ans, 3^ème^ geste, 2^ème^ pare. Le seul facteur de risque de grossesse ectopique retrouvé chez elle était un antécédent de contraception par dispositif intra-utérin. La symptomatologie clinique était dominée par la survenue de douleurs pelviennes sur une aménorrhée de 5 semaines. Le dosage des BHCG fait aux urgences était de 2134 UI/L. Le diagnostic de grossesse ovarienne a été évoqué en préopératoire suite à la visualisation en échographie d'une image arrondie faisant 26mm avec double couronne hyperéchogéne au niveau de l'ovaire gauche sans épanchement libre associé au niveau du cul de sac de douglas. Le traitement de cette grossesse avait comporté une cœlioscopie avec exérèse des villosités choriales ectopiques de siège ovarien gauche. Un traitement médical par méthotrexate à raison de 1mg par kilogramme de poids de la patiente en injection intramusculaire a été administré en postopératoire immédiat vu le risque de persistance d'un infime matériel trophoblastique. Le diagnostic a été confirmé par l'étude histologique de la pièce opératoire. Les suites opératoires étaient simples.

**Figure 1:**
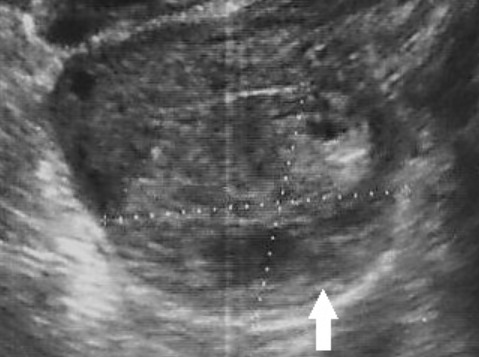
Image arrondie faisant 26mm avec double couronne hyperéchogéne au niveau de l'ovaire gauche évoquant une grossesse ovarienne (flèche)

